# Myricetin Increases Circulating Adropin Level after Activation of Glucagon-like Peptide 1 (GLP-1) Receptor in Type-1 Diabetic Rats

**DOI:** 10.3390/ph15020173

**Published:** 2022-01-31

**Authors:** Ying-Xiao Li, Kai-Chun Cheng, I-Min Liu, Ho-Shan Niu

**Affiliations:** 1 Department of Nursing, Tzu Chi University of Science and Technology, Hualien 970302, Taiwan; yxli@ems.tcust.edu.tw; 2 Department of Pharmacy, College of Pharmacy and Health Care, Tajen University, Pingtung County 90741, Taiwan; kc-cheng@tajen.edu.tw (K.-C.C.); iml@tajen.edu.tw (I.-M.L.)

**Keywords:** myricetin, adropin, opioid μ-receptor, β-endorphin, GLP-1, diabetes

## Abstract

Myricetin is a common plant-derived flavonoid, considered an agonist of glucagon-like peptide 1 (GLP-1) receptor. It improves glycemic control and helps reduce body weight in diabetic subjects. The potential mechanisms of action of myricetin in this context might be enhancing the secretion of β-endorphin (BER) to activate peripheral μ-opioid receptors. Moreover, adropin is a nutritionally regulated peptide hormone, which regulates energy metabolism, and plays a role in ameliorating diabetes. Because their mechanisms of insulin sensitivity are closely related, we hypothesized that myricetin may interact with adropin and plasma BER. The present study investigated the glucose-lowering effect of acute and chronic treatments of myricetin in type-1 diabetic rats. Plasma BER and adropin levels were determined by enzyme-linked immunosorbent assay (ELISA). The secretion of BER was measured in rats who received adrenalectomy. The changes in adropin gene (*Enho*) or mRNA level of GLP-1 receptor were measured using qPCR analysis. The results showed that myricetin dose-dependently increased plasma BER and adropin levels like the reduction of hyperglycemia after bolus injection as acute treatment. In addition, these effects of myricetin were inhibited by the antagonist of GLP-1 receptor. Moreover, in HepG2 cell line, myricetin induced GLP-1 receptor activation, which modulated the expression of adropin. In diabetic rats, the plasma adropin increased by myricetin is mainly through endogenous β-endorphin after activation of GLP-1 receptor via bolus injection as acute treatment. Additionally, chronic treatment with myricetin increased adropin secretion in diabetic rats. In conclusion, our results provide a new finding that activation of opioid μ-receptor in the liver may enhance circulating adropin in animals.

## 1. Introduction

Myricetin, a natural phenolic compound, is present in fruits, vegetables, tea, berries, red wine, and medical plants [1]. In patients with type-2 diabetes (T2DM), myricetin has been observed to reduce plasma glucose levels [2,3], to protect pancreatic β-cells and restores islet function [4]. It also has protective effects on diabetic cardiomyopathy [5]. The essential mechanism of myricetin for improving insulin sensitivity might be the amelioration of impaired signaling intermediates downstream of insulin receptors through enhancing the secretion of β-endorphin (BER), which in turn leads to the activation of peripheral μ-opioid receptors [6]. Additionally, myricetin has been identified as an agonist of glucagon-like peptide 1 (GLP-1) receptor using receptor knock-out mice [7]. However, myricetin also increased GLP-1 due to inhibition of degrading enzyme dipeptidyl peptidase-4 (DPP-4) at a higher dose in rats [3]. Therefore, activation of GLP-1 signaling in myricetin-induced action seems important.

Polyphenols also ameliorate lipid profiles [8]. Myricetin may reduce body weight gain, in addition to the level of blood lipids and adipocyte size in obese rats fed a high-fat diet (HFD) as has been demonstrated [9]. Interestingly, a parallel increase in plasma BER and adropin was observed in myricetin-treated obese rats [10]. Flavonoids may induce white adipose tissue browning and activate brown adipose tissue to increase energy consumption for inhibiting weight gain and preventing metabolic diseases [11]. 

Adropin is a peptide expressed in many tissues, particularly in the liver and the brain. It is encoded by the energy homeostasis associated (Enho) gene [12]. Adropin is involved in controlling metabolism and energy homeostasis [11]. In diabetic rats, hepatic adropin expression increases with oxidative stress [13]. In addition, adropin levels correlate with endothelial dysfunction in cardiovascular diseases and enhanced disease progression [14]. A possible mechanism is adropin association with signal transducer and activator of transcription 3 (STAT3) activation [15]. A recent study showed that STAT3 signaling contributed to the protective effects of GLP-1 expression [16]. Therefore, it is interesting to investigate the correlation between adropin and myricetin in glucose regulation. Moreover, an association of BER and adropin elevated by myricetin has remained unclear. 

In the present study, we applied a diabetic animal model to investigate the difference of myricetin-induced changes in BER and adropin between acute and chronic treatments. The obtained results provide new findings that are useful to explore the linkage of GLP-1 and adropin in the metabolic effects of myricetin.

## 2. Results

### 2.1. Effects of Myricetin on Plasma Glucose, Beta-Endorphin (BER), and Adropin Levels in Type-1 Diabetic Rats

**Figure 1:**
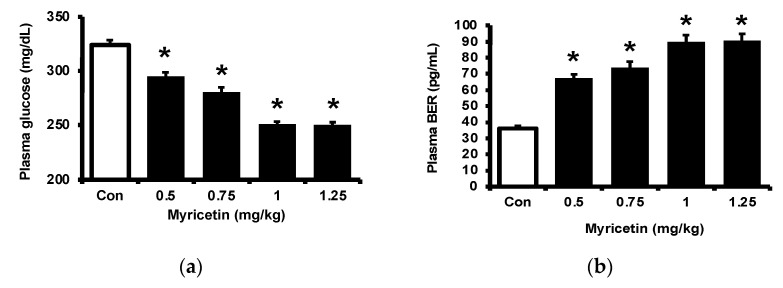

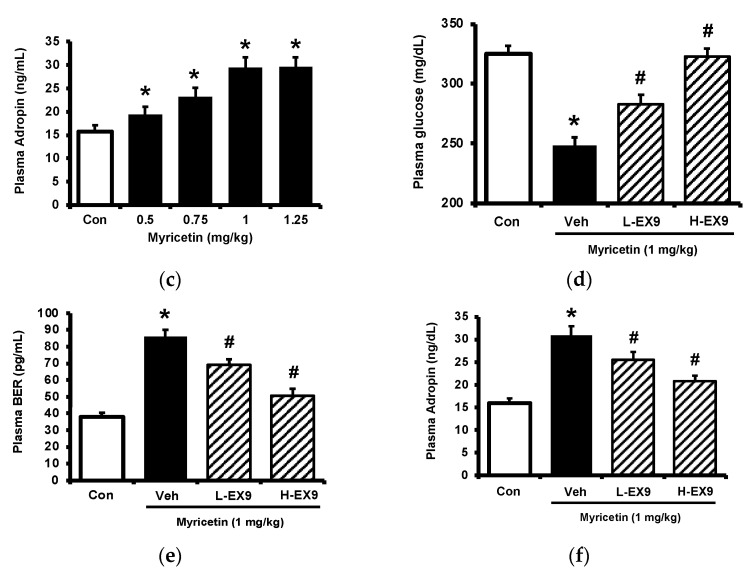
Effects of bolus injection of myricetin on plasma parameters in type-1 diabetic rats. (**a**) plasma levels of glucose, (**b**) plasma levels of BER, (**c**) plasma levels of adropine in type-1 diabetic rats receiving myricetin in the indicated concentrations. Blockade of myricetin-induced action by Ex9 regarding the changes in (**d**) plasma levels of glucose, (**e**) plasma levels of BER, and (**f**) plasma levels of adropin in type-1 diabetic rats receiving myricetin treatment. The results in each column are shown as the mean ± SEM (*n* = 8 per group). * *p* < 0.05 vs. the diabetic group without any treatment; # *p* < 0.05 vs. the diabetic group treated with myricetin. Abbreviation: BER, beta-endorphin; Con, control; Veh, vehicle; Ex9, exendin (9–39); L-Ex9, low-dose of exendin (9–39); H-Ex9, high-dose of exendin (9–39).

Consistent with our previous report [17], myricetin may attenuate the hyperglycemia in streptozotocin (STZ)-induced diabetic rats, as shown in Figure 1a. Also, plasma BER levels were dose-dependently increased by myricetin (Figure 1b). Notably, the adropin level in hyperglycemia was markedly raised by myricetin in the same doses (Figure 1c). Then, we used exendin 9–39 (Ex9) to block the GLP-1 receptor in rats as described in the previous report [18]. In the presence of Ex9, the acute effects of myricetin were dose-dependently reduced, including the decrease of hyperglycemia (Figure 1d), the higher plasma BER (Figure 1e) and increased plasma adropin levels (Figure 1f). Therefore, activation of the GLP-1 receptor seems important in these effects of myricetin.

### 2.2. Activation of GLP-1 Receptor by Myricetin

**Figure 2:**
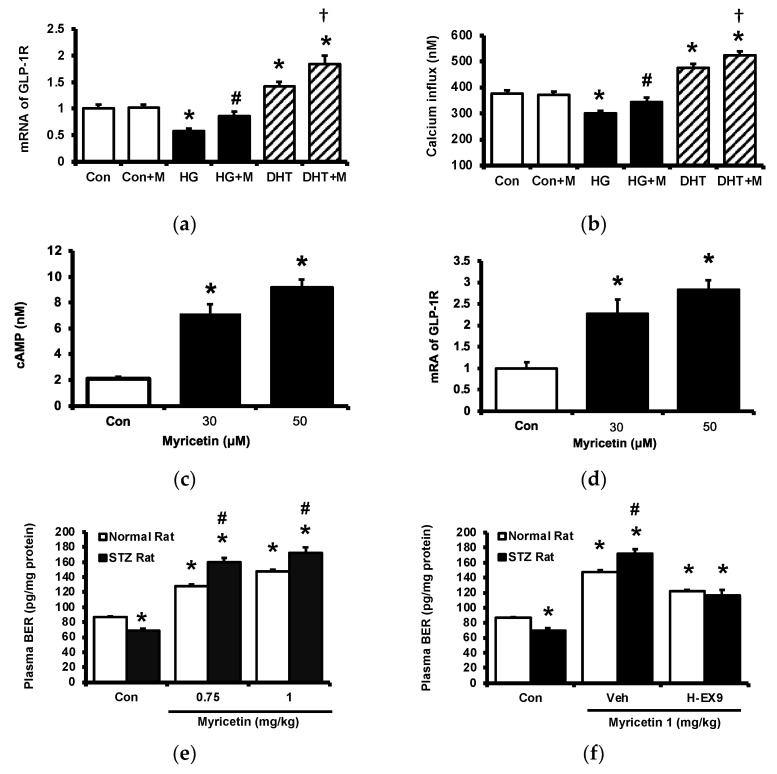
The effects of myricetin on cAMP accumulation, the expression of GLP-1 receptors and the palsma BER level in hyperglycemic conditions. Changes of (**a**) mRNA levels of GLP-1 receptor or (**b**) intracellular calcium levels in PC12 cell incubated with myricetin (1 μM) for 60 min under the conditions of high glucose or DHT exposure; (**c**) the changes of cellular cAMP concentrations, and (**d**) mRNA expression levels of GLP-1 receptor in H9c2 cells receiving myricetin tratment at indicated concentration for 24 h. The effects of myricetin on BER secretion were compared in isolated adrenal medulla. (**e**) Myricetin dose-dependently increased BER secretion from isolated adrenal medulla tissue of diabetic rats. (**f**) Ex9, an antagonist of GLP-1 receptor, dose-dependently inhibited the effect of myricetin on BER secretion. The results in each column are shown as the mean ± SEM (*n* = 8 per group). * *p* < 0.05 vs. the normal control group without any treatment. # *p* < 0.05 vs. the normal rats (white column). † *p* < 0.05 vs. the DHT group. Abbreviation: BER, beta-endorphin; DHT, dihydrotestosterone; Con, control; Veh, vehicle; H-Ex9, high-dose of exendin (9–39).

Cultured PC-12 cells were incubated with myricetin. One group of PC-12 cells cultured in a hyperglycemic medium was used to reduce the expression of GLP-1 receptor and another group was pretreated with dihydrotestosterone (DHT) to promote the expression of GLP-1 receptor in PC-12 cells. The GLP-1 receptor gene expression in each group was shown in Figure 2a, the expression of GLP-1 receptor was reduced in PC-12 cells cultured under high glucose medium and DHT promoted it more markedly than the control group. Then, the increase of cellular calcium level induced by myricetin in each group was determined. Interestingly, myricetin increased cellular calcium content depending on the expression level of the GLP-1 receptor in PC-12 cells (Figure 2b). Moreover, myricetin also dose-dependently increased cAMP levels in cultured cardiomyocytes H9c2 cells (Figure 2c). The mRNA level of the GLP-1 receptor in H9c2 cells was also raised by myricetin in the same manner (Figure 2d). In the isolated adrenal medulla, myricetin increased the secretion of BER that was more sensitive in tissues isolated from type-1 diabetic rats than that from the normal rats (Figure 2e). Additionally, this effect of myricetin was markedly blocked by Ex9 in both tissues (Figure 2f). These in vitro data all support the mediation of the GLP-1 receptor in myricetin-induced actions.

### 2.3. Circulating Adropin and Beta-Endorphin (BER) Levels Changed by Acute Treatment with Myricetin in Type-1 Diabetic Rats

**Figure 3:**
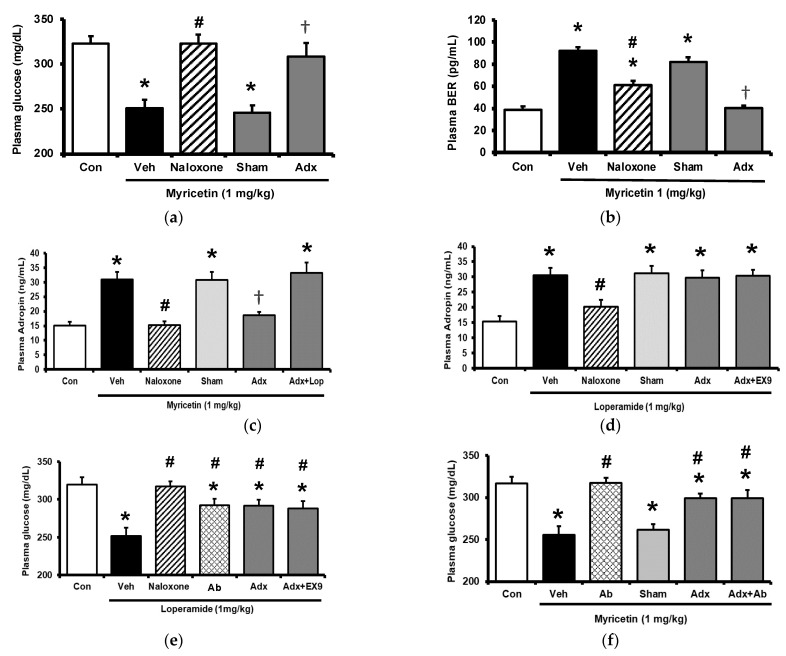
Effects of myricetin in rats receiving adrenalectomy or the treatment of anti-adropin antibodies. (**a**) The effects of myricetin on (**a**) blood glucose levels, (**b**) plasma BER levels in diabetic rats and adrenalectomized rats; (**c**) plasma levels of adropin in diabetic rats and adrenalectomized rats with or without loperamide treatment; the effect of loperamide on (**d**) plasma adropin levels, (**e**) blood glucose levels in diabetic rats with or without naloxone treatment, or the adrenalectomized rats administered with or without Ex9; (**f**) after the treatment of anti-adropin antibodies, the effects of myricetin on blood glucose levels in diabetic rats receiving adrenalectomy or not. The results in each column are shown as the mean ± SEM (*n* = 8 per group). * *p* < 0.05 vs. the diabetic group; # *p* < 0.05 vs. the diabetic group treated with myricetin or loperamide; † *p* < 0.05 vs. the sham group. Abbreviation: BER, beta-endorphin; Con, control; Veh, vehicle; Ab, antibody; Adx, adrenalectomy; Ex9, exendin (9–39).

Circulating adropin was increased by myricetin in parallel with the change in plasma BER in type-1 diabetic rats shown above. To understand the association between adropin and BER, the adrenal gland in rats was surgically isolated because the adrenal gland has been postulated as the main source of BER in type-1 diabetic rats [17]. The role of adropin in hypoglycemic response was investigated using pretreatment with the antibodies against adropin (Peninsula Laboratories, Belmont, CA, USA) as described above. The antihyperglycemic effect of myricetin was markedly reduced by removal of adrenal gland same as the pretreatment with naloxone in type-1 diabetic rats as shown in Figure 3a. Similarly, the increase in plasma BER by myricetin was also changed in the same way except the failure of naloxone pretreatment (Figure 3b). However, plasma adropin increase by bolus injection of myricetin was changed same as antihyperglycemic effect in these diabetic rats lacking adrenal gland or not (Figure 3c). Notably, as shown in Figure 3d, loperamide increased plasma adropin level in diabetic rats lacking their adrenal gland and this effect was also blocked by naloxone. Additionally, loperamide attenuated hyperglycemia in diabetic rats with adrenal glands or not (Figure 3e) and this effect was blocked by naloxone pretreatment. However, both effects of loperamide were not changed by Ex9 at the dose used to block the GLP-1 receptor. A direct effect of loperamide on the liver via opioid receptor activation to increase circulating adropin can thus be considered. Moreover, blockage of adropin using antibodies also reversed the hypoglycemic response to myricetin in diabetic rats lacking adrenal gland or not (Figure 3f). An effect of medication with adropin in the reduction of hyperglycemia by myricetin can thus be identified. Collectively, bolus injection of myricetin may activate the GLP-1 receptor to induce BER secretion from the adrenal gland for the elevation of circulating adropin in type-1 diabetic rats.

### 2.4. Circulating Adropin and Beta-Endorphin (BER) Levels Changed by Chronic Treatment with Myricetin in Type-1 Diabetic Rats

**Figure 4:**
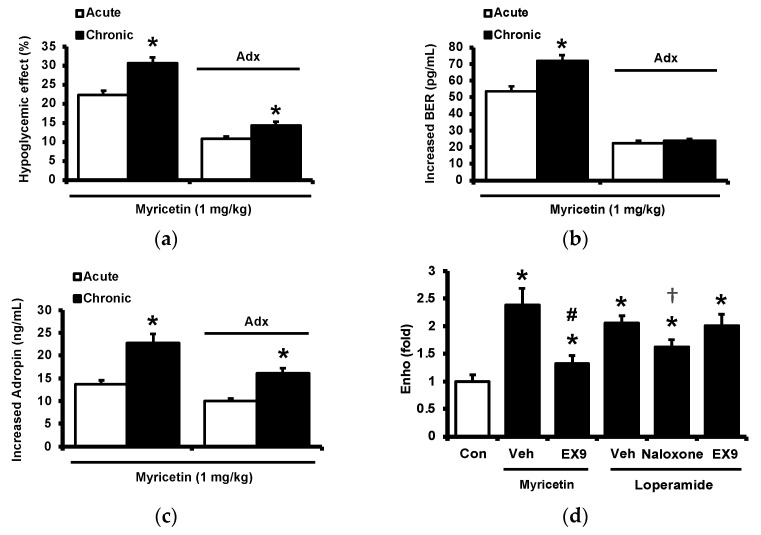

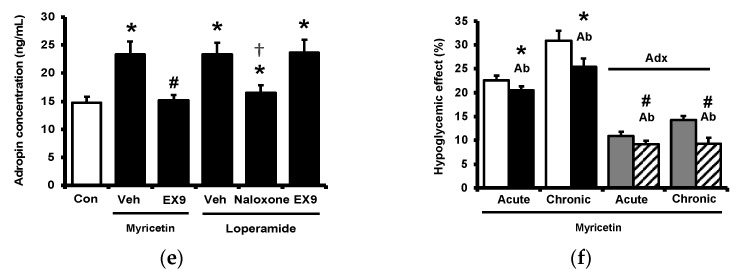
Changes of plasma parameters after acute and chronic treatment of myricetin in type-1 diabetic rats. Myricetin were administered for 4 weeks. The acute and chronic effects of myricetin on (**a**) the hypoglycemia effect and (**b**) plasma BER levels, (**c**) plasma adropin levels have been measured in diabetic rats or diabetic rats receiving adrenalectomy. In HepG2 cells, (**d**) the expression of Enho gene, and (**e**) adropin protein levels was promoted by myricetin after incubation for 24 h. Ex9 treatment ameliorated these effects. Furthermore, loperamide increased adropin expression at the mRNA and protein levels. Naloxone inhibited these effects by blocking opioid receptors. (**f**) Hypoglycemic response to myricetin after chronic treatment in diabetic rats lacking adrenal gland was markedly reversed by adropin antibodies. The results in each column are shown as the mean ± SEM (*n* = 8 per group). * *p* < 0.05 vs. the diabetic control group; # *p* < 0.05 vs. the diabetic group treated with myricetin; † *p* < 0.05 vs. the diabetic group treated with loperamide. Abbreviation: BER, beta-endorphin; Con, control; Veh, vehicle; Ab, antibody; Adx, adrenalectomy; Ex9, exendin (9–39).

Diabetic rats which received a daily injection of myricetin at 1 mg/kg for 4 weeks were used to investigate the chronic effects of myricetin. Differences in plasma glucose and biomarkers between chronic effect and acute effect of myricetin were compared. As shown in Figure 4a, the hypoglycemic activity of myricetin was more marked after chronic treatment than bolus injection in type-1 diabetic rats. In the absence of the adrenal gland, hypoglycemic effects of myricetin were reduced but not abolished in a way less market than acute treatment. Interestingly, increased plasma BER in chronic treatment was less than bolus injection of myricetin although both were disappeared during the removal of the adrenal gland (Figure 4b). Moreover, the circulating adropin increase in diabetic rats by chronic treatment with myricetin was more significant than that observed after bolus injection (Figure 4c). Increased plasma adropin by myricetin was still observed in diabetic rats lacking the adrenal gland after chronic treatment. A direct effect and/or opioid-independent effect of myricetin on adropin secretion thus seems possible. This view was further supported by using cultured hepatocytes, which are known as the main source of circulating adropin [11]. In HepG2 cells, the human hepatoma cells, the expression of Enho gene was significant increased by myricetin, which was inhibited by Ex9 at the dose for blockade of the GLP-1 receptor. Moreover, the incubation of loperamide up-regulated Enho gene expression (Figure 4d) and the protein concentration of adropin (Figure 4e), which were blocked by naloxone (the inhibitor of opioid receptor) or Ex-9 (the inhibitor of GLP-1 receptor) at the effective concentration, respectively. Otherwise, the hypoglycemic activity of chronic myricetin treatment in diabetic rats lacking adrenal gland was attenuated by preteated of anti-adropin antibodies. The inhibit effect of antibodies was more significant in chronic treatment than in bolus injection of myricetin (Figure 4f).

## 3. Discussion

In the present study, we found that myricetin may enhance adropin secretion after activation of the GLP-1 receptor. Notably, myricetin increases circulating adropin levels mainly through endogenous opioids after bolus injection in type-1 diabetic rats while direct stimulation of adropin secretion by the chronic treatment of myricetin.

Myricetin is a plant-derived polyphenol in the glycoside form (O-glycosides) found in vegetables, fruits, nuts, berries, and many medicinal herbs [19]. In vitro, myricetin exerted both a potent antioxidant and a pro-oxidant effect. Myricetin has many beneficial effects, which include potential antimicrobial, antioxidant, immunomodulatory and cardioprotective activity, and so on [19]. Myricetin could activate GLP-1 receptor expressions in the GLP-1 receptor-knockout model [7]. Our results indicated that myricetin dose-dependently increased the GLP-1 receptor expression in PC-12 cells. Incubating PC-12 cells in a high glucose medium decreased the GLP-1 receptor expression [20]. The effects seem correlated with STAT3 activation [15]. Furthermore, myricetin increased cAMP levels and promoted the mRNA levels of the GLP-1 receptor [21] in H9c2 cells [22]. Ex9, an antagonist of the GLP-1 receptor, blocked the secretion of opioids from the adrenal medulla induced by myricetin [21]. Recently, myricetin has been demonstrated to inhibit DPP-4 and enhance the GLP-1 levels in type-2 diabetic rats [3]. The increase of the GLP-1 receptors induced by myricetin was markedly higher in diabetic rats administered with 20 mg/kg orally compared with that in the group, who treated with myricetin at the dose of 1 mg/kg) [7]. Moreover, myricetin at a high dose (20 mg/kg) improved the antioxidant enzyme activities and lowered the lipid peroxidation, although it was described as irrespective of its ability to restore the GLP-1 levels [3]. Multiple effects of myricetin have been reported to occur depending on the treatment dose [19]. Therefore, activation of GLP-1 receptor by myricetin seems limited to induction at low dose.

Adropin is one of the hepatokines [23] and it is known as an energy regulator of lipid and glucose metabolism [12]. In particular it has been proposed as a fat-burning hormone [11]. It is encoded by energy homeostasis-associated gene (enho) and expressed mainly in the liver and brain, in addition to its presence in muscle, heart, pancreas, and kidneys [24]. Plasma adropin was reduced in subjects with obesity and/or insulin resistance, and loss of body weight led to an increase in circulating adropin levels [25]. In addition to endothelial regulation [26], a negative correlation of adropin with arterial blood pressure supports the action of adropin as a vascular protector [27]. Therefore, adropin has autocrine/paracrine roles in peripheral tissues [28]. Treatment with liraglutide to activate the GLP-1 receptor may assist the improvements in diabetic patients due to an increase in adropin levels [29]. In the current study, we provide a new view that the activation of the GLP-1 receptor by myricetin increased the circulating adropin in type-1 diabetic rats. 

Opioid-mediated myricetin-induced antihyperglycemic effect has been demonstrated in type-1 diabetic rats [17]. It has also been mentioned in other herbal extracts [30], mainly through activation of opioid mu-receptors [31]. The merits of opioid (BER) in the regulation of glucose homeostasis were indicated for a long time including the insulin secretion [32] or not [33]. Recently, liraglutide has become known to induce opioid secretion after activation of the GLP-1 receptor in type-1 diabetic rats [34]. Our results are consistent with this, except for the fact myricetin was a chemical agonist. No report has mentioned the induction of adropin secretion by opioids. In the present study, we demonstrated that activation of opioid mu-receptor may promote adropin secretion both in vivo and in vitro. Collectively, myricetin may increase circulating adropin mainly through BER secretion after activating the GLP-1 receptor in type-1 diabetic rats receiving bolus injection shown as acute treatment. Additionally, the direct effect and/or opioid-independent effect of myricetin on adropin secretion may involve in these diabetic rats after a repeated treatment of myricetin. These findings were also not mentioned before. 

In cultured human hepatoma cells, HepG2 cells, myricetin may promote Enho gene and adropin levels. This in vitro effect of myricetin was also blocked by Ex9 at the dose effective to block the GLP-1 receptor. The same results were also induced by loperamide in HepG2 cells. The direct effect of loperamide on hepatocytes is no doubt due to the presence of opioid receptors [34]. However, the GLP-1 receptor was initially considered as not detectable in the liver [35]. Later, it has been identified GLP-1 receptor in human hepatocytes [36] in addition to the liver of ob/ob mice [37]. Therefore, the effect of myricetin on the adropin gene via GLP-1 receptor in HepG2 cells seems consistent with these reports. Recently, direct inhibition of hepatic glucose output by exenatide, peptide analog of GLP-1, was demonstrated to mediate through Fibroblast Growth Factor 21 (FGF21), another hepatokine [38]. However, some researchers still support the absence of GLP-1 receptors in the liver [35]. For this controversial point, multiple targets of GLP-1 analogs have been summarized to explain the reason for improvements in fatty liver and./or steatosis [39]. The indirect reduction of hepatic lipid accumulation by activation of the GLP-1 receptor has also been demonstrated [39]. Interestingly, the metabolites or degraded products of GLP-1 in livers, including GLP-1 (28–36) amide and GLP-1 (32–36) amide, have been speculated to mediate the direct hepatic effects of GLP-1 analogs through the GLP-1 receptor-independent pathway [40]. However, it remains obscure regarding the role of GLP-1 receptor in liver of animals. Therefore, details of adropin secretion from liver by myricetin in diabetic rats shall be investigated in the future. 

## 4. Materials and Methods

### 4.1. Materials

Myricetin (95%) was obtained from Acros Organics (Thermo Fisher, Geel, Belgium). Exendin 9–39 (Ex9), loperamide and naloxone and other chemicals or reagents were purchased from Sigma-Aldrich (St. Louis, MO, USA).

### 4.2. Animal Model

Male Sprague–Dawley (SD) rats weighing 260 to 290 g and obtained from the National Laboratory Animal Center (Taipei, Taiwan) were used. Overnight fasted rats received an intravenous injection of STZ (65 mg/kg) were applied to induce the model as described previously [17]. One week later, STZ-treated rats were considered as diabetic once they showed the plasma glucose over 300 mg/dL. Then, experiments were performed within 2 weeks after diabetes onset. To minimize animal suffering, rats were anaesthetized with intraperitoneal injection of sodium pentobarbital (35 mg/kg) before the procedures. All experimental procedures performed in studies involving animals were approved by the Local Ethics Commission for Animal Experiments of Tajen University (IACUC 106-21) and were in accordance with the 1996 NIH Guide for the Care and Use of Laboratory Animals.

### 4.3. Experimental Protocol

Diabetic rats randomly distributed into eight equal groups were arranged to receive the acute or chronic treatment with myricetin that was dissolved in 70% ethanol as stock solution [17]. 

In a preliminary experiment, subcutaneous injection (SC) of myricetin attenuated the hyperglycemia same as that induced by intravenous injection (iv) in type-1 diabetic rats described in previous report [6]. Therefore, SC injection of myricetin at effective dose was used in current study and bolus injection was indicated as the acute treatment. Pharmacological inhibitor was pretreated by intravenous injection at 30 min before the injection of myricetin in each. Antibodies against adropin (MAB9690, bio-techne, Minneapolis, MN, USA) at 1:2500 dilution [41] were treated in same manner to investigate the role of adropin. In fasting diabetic rats received bolus injection of myricetin, blood samples (0.1 mL) were collected from the tail vein under anesthesia for measurement of plasma glucose and plasma biomarkers levels as described below. 

Additionally, the chronic effects of myricetin on plasma glucose and biomarkers after daily repeated injection were also investigated. Other groups of diabetic rats received daily injection of myricetin at same dose for 4 weeks were used as the chronic effects of myricetin. In fasting diabetic rats received chronic treatment with myricetin, blood samples were also collected from the tail vein in same manner to estimate the plasma glucose and plasma biomarkers levels. Variations between chronic effect and acute effect were then compared. For easier understanding, changes in fasting plasma glucose were compared using the hypoglycemic activity (%) of myricetin as described previously [18]. Difference in plasma biomarkers was indicated as the increased amount of each using the values between before and after of myricetin treatments. 

### 4.4. Laboratory Determinations

Plasma glucose level was measured by the glucose oxidase method, using an analyzer (Quik-Lab, Ames; Miles Inc., Elkhart, IN, USA) as described in a previous method [17]. Biomarkers in plasma levels were determined by enzyme-linked immunosorbent assay (ELISA) using a com-mercially available kit, including β-endorphin (BER) and adropin (Peninsula Laboratories, Belmont, CA, USA). The adropin level in HepG2 cells was estimated by ELISA kit for humans (Phoenix Pharmaceuticals, Belmont, CA, USA). 

### 4.5. Adrenalectomy in Rats

Under anesthesia with sodium pentobarbital (35 mg/kg, i.p.), rats were received bilateral adrenalectomy using the dorsal approach as described previously [17]. Before the surgery, rats were fed ad libitum a standard chow and 0.9% saline to drink. Rats received the sham operation (controls) were fed in same manner. The animals appeared alert and in good health after surgery. Adrenalectomized rats received saline supplemented with corticosterone as a replacement for their drinking water for 2 weeks, which was then replaced by saline alone (gradually reduced within a week). After the recovery, rats were then induced with type 1-like diabetes following the previous method [17]. Then, they were used for experiments in each as the below.

### 4.6. Incubation of Isolated Adrenal Medulla

The adrenal glands were obtained from rats with diabetes after sacrificed, and the cortex were immediately removed as described previously [18]. Tissues were then placed in an incubator for a 15 min pre-incubation at 37 °C and bubbled with air (95% O_2_ and 5% CO_2_) under continuous agitation with 2 mL modified Krebs solution, as in our previous method [13]. Then, the tissues were transferred to fresh incubation tubes and further incubated with myricetin for another 60 min under continuous agitation (40 cycles/min). Placing the tubes on ice terminated the incubation. The incubated medium was then collected and frozen at −70 °C until the assay for beta-endorphin-like immunoreactivity (BER), as described previously [17]. Results were compared the responses to myricetin at various concentrations in adrenal medulla isolated from rats with diabetes. Antagonist specific for GLP-1 receptor (Ex9) was also used to inhibit the effect of myricetin through a 30-min pretreatment.

### 4.7. Incubation of Cultured Cells

In the present study, three kinds of cell line were used in experiments including rat adrenal pheochromocytoma cell-derived PC12 cell line (PC-12), cardiomyocytes (H9c2 Cells), and human hepatoma cell line (HepG2 cells). They were obtained from the Culture Collection and Research Center of the Food Industry Institute (BCRC) cultured for treatment as described below.

#### 4.7.1. PC-12 Cells

The rat adrenal pheochromocytoma cell-derived PC12 cell line (BCRC60048) at 2 × 106 cells/dish were cultured in RPMI medium (Gibco BRL, Grand Island, NY, USA) containing 2 mM L-glutamine, 15% horse serum, 2.5% fetal bovine serum and 0.1% gentamicin in 10-cm dishes.One group of PC-12 cells was cultured in hyperglycemic medium and the expression of GLP-1 receptor was markedly reduced as described previously [20]. Another group was pretreated with DHT to promote the expression of GLP-1 receptor following previous method [22] and it was confirmed in the present study using the analysis of mRNA levels in PC-12 cells below. Then, the changes of intracellular Ca^2+^ level stimulated by myricetin for 60 min in two groups of PC-12 cells were compared with that in the regular cultured PC-12 cells using as normal control. Fura-2-acetoxymethyl ester (fura-2 AM) was used as fluorescent indicator to determine the intracellular Ca^2+^ level in the above three groups of PC-12 cells. The peak excitation wavelength changes from 380 nm to 340 nm were recorded using a fluorescence spectrofluorometer (F-2000; Hitachi, Tokyo, Japan). The intracellular calcium [Ca^2+^]_i_ values were evaluated following our previous protocol [22]. Background autofluorescence obtained from untreated cells was subtracted from all measurements.

#### 4.7.2. H9c2 Cells

H9c2 cells (BCRC No. 60096) were cultured as previously described [22]. Briefly, the H9c2 cells were maintained in Dulbecco’s modified Eagle’s medium (DMEM, pH 7.2; Gibco-BRL Life Technologies, Gaithersburg, MD, USA) supplemented with 10% fetal bovine serum. The cells were plated at 6000 cells/cm^2^ and allowed to proliferate in the medium. The medium was replaced on the second day. Then, the cells were used for the subsequent experiments because H9c2 cells show similar responses as the primary rat neonatal cardiomyocytes [22]. H9c2 cells were incubated with phosphodiesterase inhibitors (IBMX 5 µM) for 30 min, and then treated with myricetin at indicated concentration for another 1 h. Sample lysates were then collected and the intracellular cAMP levels were measured using cAMP Assay Kit (Abcam, Cambridge, MA, USA). Variations between treatment with myricetin or not were indicated as the increased cAMP in each. Additionally, changes in GLP-1 receptor expression were determined in these samples using the assay of mRNA levels in H9c2 cells as described below. 

#### 4.7.3. HepG2 Cells

HepG2 cells (BCRC No. 60025) were cultured in the Dulbecco’s modified Eagle’s medium (DMEM, GE Healthcare Life Sciences, Pittsburgh, PA, USA) containing 10% fetal bovine serum (GE Healthcare Life Sciences), 1% penicillin/streptomycin (GE Healthcare Life Sciences) under standard conditions (95% air, 5% CO_2_, 37 °C) according to previous report [15]. HepG2 cells (1 × 10^6^ cells/dish) were seeded in the 10-cm culture dishes. To investigate the effect of myricetin on change of adropin in HepG2 cells, cells incubated with myricetin at the indicated dose for 24 h were used to compare with vehicle-treated control. Inhibitor was preincubated for 30 min before the addition of myricetin. Then, same as the previous report [15], the cells were collected for assay of human Enho gene using qRT-PCR or the level of adropin by ELISA kit for humans [42]. Additionally, effect of loperamide was also investigated in same manner.

### 4.8. Quantitative Reverse-Transcription Polymerase Chain Reaction (qRT-PCR)

Total RNA from the adrenal medulla was isolated using TRIzol. The primers used in qRT-PCR were obtained from Roche (Roche Diagnostics GmbH, Mannheim, Germany). The concentration of each PCR product was calculated relative to a corresponding standard curve. Relative gene expression data were analyzed using real-time quantitative PCR and the 2-ΔΔCq method. This gene expression was shown as the ratio of the target gene level to that of β-actin, the housekeeping control, as described in the previous report [42]. Each sample was run in duplicate. The primers used were as follows:

GLP-1 Receptor

forward: 5′-AGTGCGAAGAGTCCAAGCAA-3′
reverse: 5′-TTGAGGGCAGCGTCTTTGAT-3′

Human Enho gene

forward: 5′-GTTGTCCCGCCTCTC-3′
reverse: 5′-CCACACACAGCGACTTCTTG-3′

β-Actin

forward: 5′-CATCCAGGCTGTGTTGTCCC-3′
reverse: 5′-CACGCACGATTTCCCTCTCA-3′

### 4.9. Statistical Analysis

The data were presented as means ± SEM. The hypoglycemic activity (%) of myricetin was calculated as the percentage decrease of the initial glucose value according to the formula: (Gi − Gt)/Gi × 100%, where Gi is the initial glucose concentration and Gt is the plasma glucose concentration after treatment of myricetin. The data were analyzed by one-way ANOVA and Dunnett’s post-hoc test, or two-way ANOVA and Bonferroni’s post-hoc tests. The normal distribution of data was checked using the maximum likelihood method, with the significance level set at *p* < 0.05. Results were subjected to statistical analysis using SPSS 23 (IMB Inc., Armonk, NY, USA).

## 5. Conclusions

Our data demonstrate that myricetin increased circulating adropin in type-1 diabetic rats by activating the GLP-1 receptor. The acute treatment of myricetin increased plasma adropin mainly through the up-regulated endogenous β-endorphin. Furthermore, direct stimulation of adropin secretion is also involved during the chronic treatment with myricetin in type-1 diabetic rats.

## Data Availability

The data in the present study is confidential.
